# Numerical and Experimental Assessment of Poly-Pyrrole Used in Spinal Cord Injuries

**DOI:** 10.3390/biomimetics10100677

**Published:** 2025-10-09

**Authors:** Carlos Alberto Espinoza-Garcés, Axayácatl Morales-Guadarrama, Elliot Alonso Alcántara-Arreola, Jose Luis Torres-Ariza, Mario Alberto Grave-Capistrán, Christopher René Torres-SanMiguel

**Affiliations:** 1 Unidad Profesional Zacatenco, Instituto Politécnico Nacional, Escuela Superior de Ingeniería Mecánica y Eléctrica, Ciudad de Mexico 07338, Mexico; 2 Departamento de Ingeniería Eléctrica, Universidad Autónoma Metropolitana Unidad Iztapalapa, Ciudad de Mexico 09340, Mexico

**Keywords:** poly pyrrole, elastic modulus, shear modulus, spinal cord, scaffold, COMSOL

## Abstract

Some common conductive polymers are polyfuran, polyacetylene, polythiophene, and poly-pyrrole. Since their discovery, many researchers have been exploring and evaluating their conductive and electronic properties. Various applications have been developed for conductive materials. Their biocompatibility offers a new alternative for studying and solving complex problems, such as cellular activity, or, more recently, for use as neural implants and as an alternative to spinal cord regenerative tissue. This is particularly true for the use of poly pyrrole. The main obstacle lies in estimating some of the mechanical properties, such as Young’s or shear modulus values for poly pyrrole, since these vary depending on the type of synthesis used. This article outlines a composite methodology for characterizing the elastic modulus according to ASTM D882 and the shear modulus according to E143 standards. It is specifically designed and applied for 3D composite samples involving PLA and PPy, where the PPy was processed by plasma oxidation. As a result, an increase of 360.11 MPa in the modulus of elasticity is observed on samples coated with poly pyrrole. The results are evaluated through a numerical test using COMSOL Multiphysics software 6.2 version, finding a similar behavior in the elastic zone, as indicated by the stress–strain diagram. The statistical analysis yields consistent data for tensile and shear results, with low to moderate variability.

## 1. Introduction

A traumatic spinal cord injury is defined as damage to spinal tissue; it is possible to lose temporarily or permanently motor and sensory functions. Commonly, it is occasioned by external physical impact, chronic disease, or degenerative disease [1]. It could be considered in two phases, primarily when trauma to the spine happens, resulting in fractures and vertebral dislocation. Secondary injury is produced by chemical and mechanical damage to spinal tissues, reflecting a multi-process that follows primary injury and can last for several weeks [2]. Many degrees of paralysis or sensory disorders could be the result of SCI. For example, neurogenic shock is associated with hypotension in cervical injuries, and cardiovascular disease when a complication of injuries to the autonomic nervous system is compromised [3]. There exist some treatments that incorporate components whose mechanical properties favor cell migration, such as scaffolds or biomaterials, in addition to medicaments [4]. Since the discovery of conducting polymers, many researchers have been exploring and evaluating their conductive and electronic properties. One of the most characteristic properties of these kinds of polymers is their low energy or high electron affinities [5]. Since 1977, conductive polymers have been studied with great interest. Recent research has explored many of their applications in the biomedical field. Some polymers can exist in various forms, including nanoparticles, gels, solids, or films, and their chemical or physical properties can enhance specific parameters in their biological applications [6]. Another interest in bioconductive polymers lies in their electrical properties when applied to tissue stimulation. They could have the potential to regulate cellular behaviors, such as adhesion, proliferation, alignment, and tissue regeneration [7]. Some common conductive polymers are polyacetylene, polyfuran, polythiophene, and poly-pyrrole. They can be processed at low temperatures and synthesized easily; these are the most notable advantages of these polymers [8]. Multiple applications exist for conductive materials, not only for designing mechanical pieces for medical use, but their biocompatibility also offers new alternatives for studying and implementing novel solutions to complex problems. For example, the use of poly pyrrole, a polymer synthesized chemically in 1963, has the potential to be applied in biomedical applications, as one of the most essential biosensors [9]. It could modulate cellular activities; a neuronal implant is a good example that can be applied as an alternative to regenerative spinal cord tissue, as reported in recent research [10]. Implants of poly pyrrole were manufactured by plasma oxidation; this experiment was conducted on specimens of Wistar rats. First of all, the spinal cord was sectioned at the thoracic 9 level, and a fragment of polypropylene was implanted at the injury site. During 4 to 8 weeks, the rat specimen recovered in high percentages of its motor and sensory function [11]. Additionally, it is commonly used as an electrode to transfer electrical stimuli or as a conductive scaffold in tissue engineering [12]. It is common to prepare solutions based on the oxidative polymerization of pyrrole monomer.

Oxidative polymerization can be developed using chemical, electrochemical, or ultrasonic irradiation methods, as well as other techniques [13]. The most popular method for synthesizing poly-pyrrole is chemical oxidation, which is a fast and straightforward preparation process. It involves creating a solution by combining an inorganic oxidant with a pyrrole monomer. Some examples of oxidants include hydrogen peroxide, ammonium persulfate, or perchlorate [14]. The electrochemical process is purified through vacuum distillation. It is necessary to purify the electrolyte solution and connect the electrical circuit to the electrodes. A voltage is induced in the circuit, and the polymer is deposited on the electrode [15]. The process of plasma polymerization can be carried out in a cylindrical chamber, where a shaped plasma is coupled to a flow of pyrrole monomer. The discharge chamber consists of an anode and a cathode. Pyrrole monomer vapors were allowed to enter a plasma discharge chamber at a constant flow rate. A DC source was connected across the two electrodes to generate plasma. It is possible that the films were dropped at a continuous deposition time and varying the discharge power [16].

Various processes or techniques enable the evaluation or prediction of deformation mechanisms and mechanical behavior in materials. Particularly in medical applications, finite element analysis allows the assessment of multiple scenarios and structures under specific forces or interaction conditions, also suggesting a novel tool for elucidating neuronal regeneration. This hypothesis is supported by the fact that mechanical properties, when combined with biological material and tissue, conduct the electrical pulse correctly through elements to the lesion, but compromise and interrupt this flow. However, it is necessary to define each mechanical property for the material, and each variable must be correctly used and defined. Unfortunately, in the case of materials such as poly pyrrole, many of their mechanical properties depend on the synthesis method used to produce them. Much of the research focuses on describing electrical properties, while a few others explain mechanical properties. For example, Jürgen [17] evaluates the mechanical properties of polyethylene and polypropylene composite samples. In this research, chemical synthesis was used for both polymers. A tensile test was conducted to determine the Young’s modulus, yielding a range of approximately 1.41–2.73 GPa, which depends on the mass concentration of PPy on the sample. Yujie Liu [18] presents a novel alternative for measuring the mechanical properties of ultrathin polymeric films. She used a polymer film, which was first cut into a rectangular shape. After adding water, a helium laser and HD camcorder were used to emulate an optical device. Their objective is to estimate the Young’s modulus; unfortunately, this is typically applied to polymers, such as polycarbonates. A nanoscale evaluation was developed based on Alexander’s research [19] for the elastic properties of poly pyrrole thin films. In this study, electropolymerization was used to obtain poly pyrrole at different electrical potential values. Results showed a variance of nearly 18% in the elastic modulus due to the presence of PPy. Research by Sguazzo [20], which motivated our main objective, involves developing tensile and shear tests on polypropylene and polyethylene foils at different temperatures. His aim is to establish an equilibrium stress state for samples tested using tensile and torsional methods. With the loading rate, particularly, the stress magnitude increases, while the temperature decreases. According to Oliveira [21], the most characteristic method to measure the mechanical properties of polymers synthesized by plasma-polymerized thin films is nanoindentation. Explain how it is possible to recover part of neuronal function; it is a relatively recent and complex process. To offer an alternative for clarifying and understanding neuronal reconnection, this research employs the finite element method to develop numerical simulations of spinal cord models, evaluating the mechanical behavior between the implant and spinal tissue.

As mentioned before, there is limited information on how to estimate specific mechanical properties of poly pyrrole processed by plasma oxidation. Some results describe the behavior of PPy according to mass concentration. Additionally, synthesis methods directly affect their properties. Another problem originates from poly pyrrole production; it is not possible to synthesize more than 3 cm of total length to conduct at least a pure tensile test for PPPy. Furthermore, nanoindentation results on a macro scale are not reliable for simulation purposes. These challenges hinder the fundamental understanding of injury mechanisms, which need to be evaluated through numerical simulations. For this reason, this paper presents a composite methodology to characterize the elastic modulus for PPPy according to ASTM test standards D882 and E143, featuring a special design for a 3D composite sample that involves PLA as a support element, coating of PPy, and a geometrical grip section for each torsion and tensile machine test, where PPy was processed by plasma oxidation. The primary outcome is to define Young’s and shear moduli for PPPy, identify graphical differences between stress–strain diagrams with and without PPPy, and evaluate their mechanical properties as a scaffold in spinal cord simulations.

## 2. Materials and Methods

**Figure 1:**
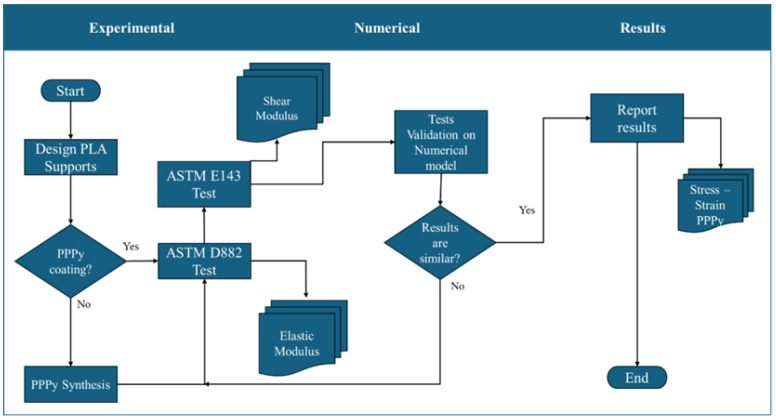
General diagram for evaluating PPPy mechanical properties.

The methodology is focused on three stages. In general terms, the first stage describes the experimental test used to establish the mechanical properties, including elastic and shear moduli, of poly pyrrole (PPPy) produced by the plasma oxidation method. Unfortunately, mass production is insufficient for designing a sample. For this reason, and as a priority, we need to use support elements. They were impressed with 3D machines, considering acid poly lactic (PLA) as the support element, with 100% fill and planar reduction of noise on the surface. The second stage focused its activities on evaluating the mechanical properties of PPPy through numerical tensile validation using 3D model simulations, and the third stage involved analyzing the results. It is essential to mention that for the first stage, 3D PLA samples are manufactured and designed according to ASTM standards for each test, as well as for the mathematical estimation of elastic modulus. Each stage is described in general terms, as illustrated in the following diagram (Figure 1).

### 2.1. Experimental Activities, First Stage

**Figure 2:**
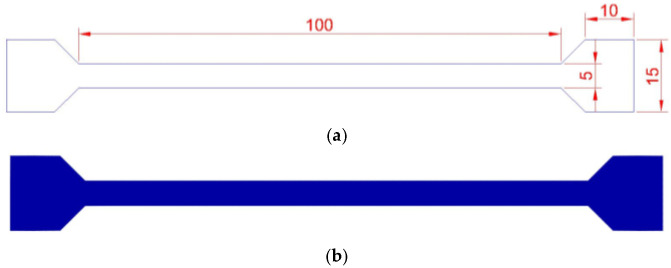
Tensile sample (**a**) dimensions and (**b**) final PLA 3D Model.

As mentioned, this stage involves considering experimental activities to obtain mechanical properties values, evaluate the stress–strain diagram differences between tensile and torsional tests on PLA samples with and without PPPy coating. Each activity is designed to meet all requirements for each test and has an order to assert and reduce error variance across all tests. First, a tensile sample was designed according to the ASTM standard test D882. Samples were then developed for tests on PLA, following the recommendations and standards. Additionally, the geometric section machine was considered for a major fix during testing. Some characteristics for sample testing are described as follows: tensile samples, according to ASTM D882 [22], have a length of a maximum of 250 mm and a minimum of 50 mm, a width of at least 5 mm and a maximum of 25.4 mm, and a thickness of less than 1 mm. For research purposes, sample dimensions are 100 × 5 × 0.5 mm (L × W × T), which is suitable for low PPy production, considering a 15 × 15 mm grip section. Figure 2 graphically illustrates all dimensions for the sample test according to ASTM standards and the final 3D model. Tensile samples are 100% full, and the configuration arguments were defined under two possible scenarios: the first scenario does not include finished support on samples or PPy coating. In contrast, the second scenario uses PPy coating.

**Figure 3:**
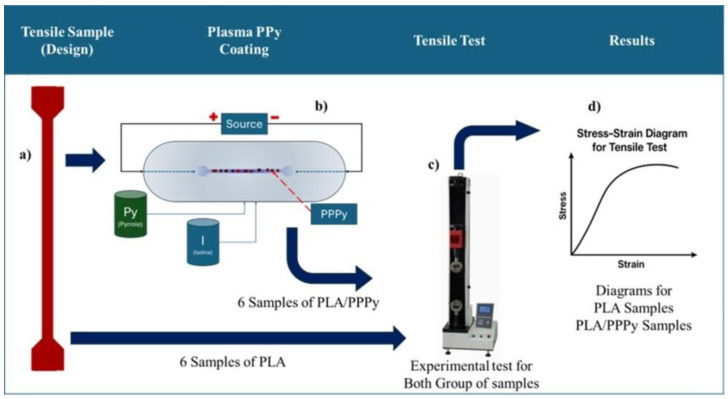
Process for Tensile Test, (**a**) Design and manufacture for tensile sample test, (**b**) Plasma process for coating PLA with PPPy, (**b**) Sample of PLA/PPPy, (**c**) Tensile test, and (**d**) Stress–Strain Diagram (results of each tensile sample test).

For the plasma oxidation method, a cylinder reactor was used. To complete the electrical circuit, PLA samples were connected at the right section (anode) and the left section (cathode). The first reactive was Py (Pyrrole) at 96% pure, as a dopant agent Iodine, 1.4 kV, 72 Pa, 180 min were the reaction conditions for voltage, pressure and reaction time to process Poly pyrrole was deposited only one side, with the intention to evaluate the homogeneity, indirectly thickness is controlled by fixing the exposure time and discharge parameters is around to 50 µm, all reactive were fed on gas, with this process is possible synthetized and at the same time coat PLA samples. Tensile test, according to the ASTM D882 Standard Test Method for Tensile Properties of Thin Plastic Sheeting, is primarily intended to estimate the elastic modulus of thin films. It consists of validating the material film thickness, explicitly specifying that this thickness must be less than 1 mm. This also defines the minimum number of samples required for testing: a minimum of 5 for isotropy and 10 for anisotropy; if sufficient material is not available, these can be reduced to 3 and 6, respectively. For the tensile test, 12 PLA samples were considered, divided into two groups: 6 samples for pure PLA and 6 samples for PLA with PPPy coating, with a coating thickness of 50 µm. Speed can be estimated based on the separation between the grips; for this study, the analysis was performed at 2 mm/min for both groups of samples with and without poly pyrrole coating. Tests were performed on the tensile testing machine, aligning the surface outside the cross-section toward the grips. The testing machine also records load, displacement, and time data in a text file, which is later exported to a data sheet for greater control and operation. To build the stress–strain diagram, an estimated stress value is calculated relative to the cross-sectional area, which is 2.5 mm^2^, by dividing the sample width (5 mm) by its thickness (0.5 mm). Figure 3 illustrates the processes for the tensile tests on PLA and PLA/PPPy samples.

**Figure 4:**
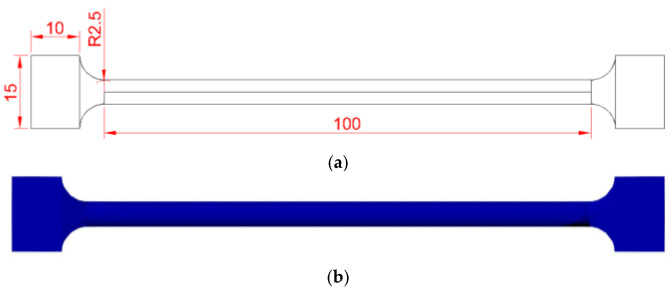
Torsion sample, (**a**) dimensions and (**b**) final PLA 3D model.

For the torsion sample design, all dimensional requirements were considered in accordance with the ASTM E 143 standard [23]. It is established that for good material selection, the material must be free from imperfections, its geometry must be cylindrical or tubular, and it must have a constant ratio. At least two diameters should be specified, one as the length and the other as a complement, to reduce alignment mistakes. The grip section on the sample was designed following the grip machine section. A total analysis length of 100 mm was established, with a 5 mm ratio and a 15 mm square grip section. For the same purpose, PLA was used as the support material for evaluating the shear modulus. All these dimensions and their final manufacture are described in Figure 4.

**Figure 5:**
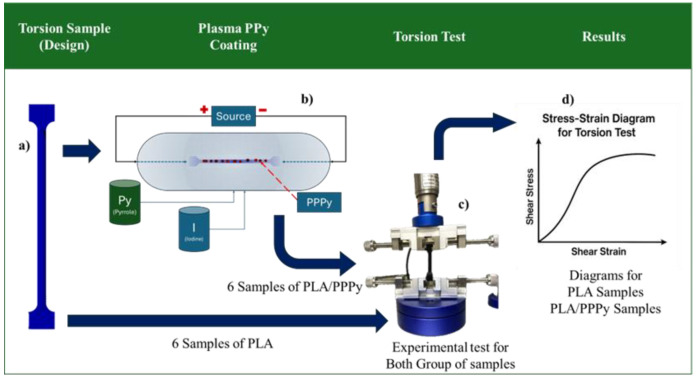
Torsion test process, (**a**) Design and manufacture for sample torsion test, (**b**) Plasma process for coating PLA with PPPy, (**b**) Torsion sample of PLA/PPPy, (**c**) Torsion test, and (**d**) Shear Stress—Strain Diagram (results of each torsion sample test).

Similarly, 12 samples form the total group for all Torsion tests; 6 of them are part of the PLA group, and the last 6 were processed like the tensile group to integrate 50 µm of PPPy. After all groups were identified, each test was developed according to the four essential steps outlined in the ASTM E 143 standard to achieve a successful test. The first of them consists of measures to determine the length of the transversal section. The alignment sample test is the next step; it is crucial to position it at the center of the grip section. For configuration machines, it is essential to establish a high angular velocity; for each test, 100°/min was defined as the test velocity. All torsion tests were conducted on an NG-203 Guangdong torsion machine. Some characteristics of this machine include a maximum torque of 500 kgf·cm and a velocity range of 6–1500°/min. The final step is to record an advertisement about temperature variance during the test; it is essential to record all temperatures. Figure 5 illustrates each step in a successful torsional test.

There are mathematical expressions that can be used to estimate the value of specific mechanical properties for torsion tests. The most important involves shear stress and Strain, just as shown in the following mathematical expressions:

(1) $$\tau =\frac{Tr}{J}\;$$

where τ = Shear stress (MPa), *T* is the value for torque in (N m), r is the radius of the sample (m), and J is the inertia polar momentum (m^4^).

To estimate Strain, it is possible to evaluate it with a mathematical expression (2).

(2) $$\gamma =\frac{r\theta }{L}\;$$

where γ = Strain (m/m), r is the radius of the sample (m), θ is the angle (rad), and L is the total sample length (m).

### 2.2. Numerical Validation for Mechanical Properties

Numerical simulation, carried out through finite element analysis, offers numerous facilities and advantages. Its approximation to reality predicts similar performance, at a lower cost, or by evaluating scenarios that compromise the physical integrity of a living being. However, like any other test, it is necessary to gather as much information as possible about the variables involved. This is particularly true when validating a material’s properties and mechanical behavior. To approach and evaluate the results of each test, numerical tensile and torsion simulations were developed using the computational tool COMSOL Multiphysics, based on 3D sample models and elasticity and shear modules obtained from torsion and tension tests, respectively.

Tensile and torsion tests are considered static analyses, and each test involves a particular process that requires specific commands or lines to yield accurate results. For tensile tests, at least four steps are necessary: importing geometry, defining the material and boundary conditions, and post-processing the data. In general terms, each step is defined as follows:

(A) Import and mesh model, *a* tensile model was incorporated into the computational tool. According to the submenu “Geometry,” it is possible to import the model. Then, with the mesh section, it is necessary to select all geometry to discretize on a fine mesh, confirmed by 4259 tetrahedral elements with an average element size of 1.1 mm.
(B) Boundary conditions are defined as follows: the grip sections are characterized by fixed and mobile geometries. The left edge of the grip section is described as having fixed geometry on lateral edges, while the right edge is defined as having mobile geometry, considering a 2 mm displacement in the X direction.
(C) Material: COMSOL integrates multiple materials into its database, including PLA and its compositions. In this case, PLA with 0–90% fill was sectioned. After, some properties were changed according to experimental results.
(D) To obtain accurate results, It is essential to process each plot to reconstruct a numerical test stress–strain diagram. It is necessary to select two volume plots; for the first, its properties must include tensile von Mises stress, and for the second plot, resultant deformation. It is possible to export all tensile results to a data sheet; however, it is sufficient to take values for the graphical section for stress and strain results.

**Figure 6:**
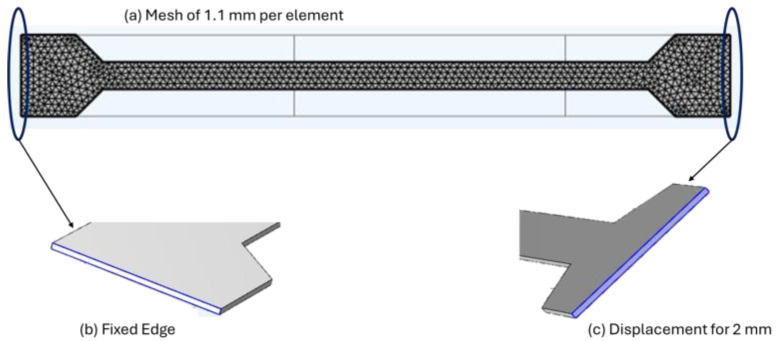
Numerical tensile simulation, (**a**) Mesh quality, (**b**) Fixed geometry on left edge, and (**c**) Prescribed displacement of 2 mm on the right edge.

As an example of the simulation process in previous tension tests, Figure 6 describes the meshing, material properties, and boundary conditions.

**Figure 7:**
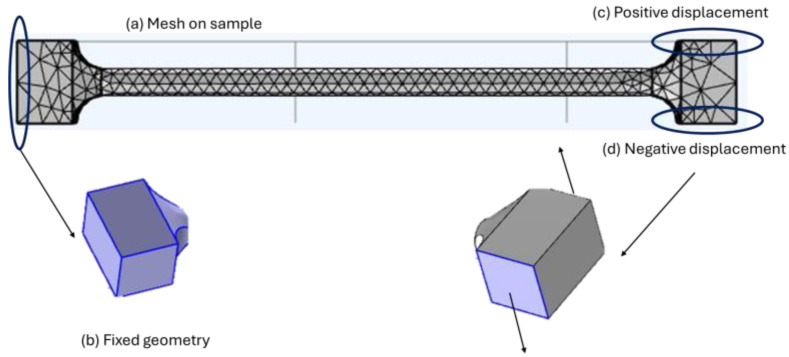
Numerical tensile simulation, (**a**) Mesh quality, (**b**) Fixed left grip geometry, (**c**) Prescribed positive displacement of 2 mm on right edge and (**d**) Prescribed negative displacement of 2 mm on counter edge.

The process for torsion testing is similar. First, it is necessary to import the torsion model into the computational tool. The mesh is then automatically discretized, defining 1959 tetrahedral elements with a 1.2 mm size on the transversal section and a 2.2 mm size on the grip sections. For boundary conditions, the left grip section was defined as fixed geometry. For the mobile section, two lateral edges were defined with a simultaneous vertical displacement, considering the conversion from angular to linear velocity (100 degrees/min, 0.1454 mm/s with a ratio of 2.5 mm). Each result from the last test involves the way to estimate shear modulus, just as the tensile test is to be analyzed graphically. The differences are that they are expressed as a tensor component for the XZ plane for stress and Strain. Figure 7 describes each condition for the torsion numerical test.

Some computational tools need more specific information about material and mechanical behavior. It is possible to achieve results with better precision if we can configure this information. With both moduli, it is possible to estimate the value of the Poisson ratio. For this process, it is necessary to involve mathematical expression 3:

(3) $$G=\frac{E}{2(1+v)}$$

where G is the Shear Modulus [MPa], E is the Elastic modulus [MPa], and v is the Poisson ratio.

If this expression is changed in the function for “v” as an unknown, it is possible to estimate this parameter so that this mathematical expression will be:

(4) $$v=\frac{E}{2G}-1$$

Unfortunately, this expression is only possible when the material is considered ideal, isotropic, homogeneous, and continuous, among other conditions. For numerical purposes, some values are reported in the literature, and the range for the PLA poison ratio is 0.35–0.40.

For each numerical test, PLA and PLA/PPPy results from experimental tests were used to evaluate and validate the mechanical behavior. Table 1 integrates the value of properties that can be configured on a computational tool.

### 2.3. Statistical Data

As a way to validate and evaluate data reliability a simple statistical process was developed, the variability coefficient value define the percentual reliability of experimental data; if this value is minor to 10% exist low variability and most of values are consistent, in the 10% to 20% exist moderate variability and final if this value is principal than 20% the variability is high as a consequence most values are inconsistent.

## 3. Results

**Figure 8:**
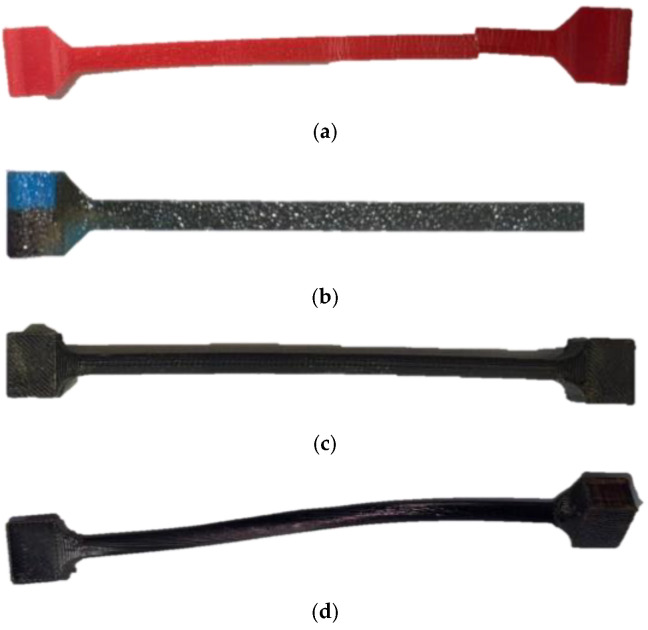
Samples: (**a**) Tensile PLA, (**b**) Tensile PLA/PPPy sample, (**c**) Torsion PLA sample, and (**d**) Torsion PLA/PPPy sample.

The surface obtained from the 3D printing of the sample models for tension and torsion tests facilitated the adhesion of the PPPy coating. Figure 8 shows the PLA model and its coating after the tension or torsion test was performed.

**Figure 9:**
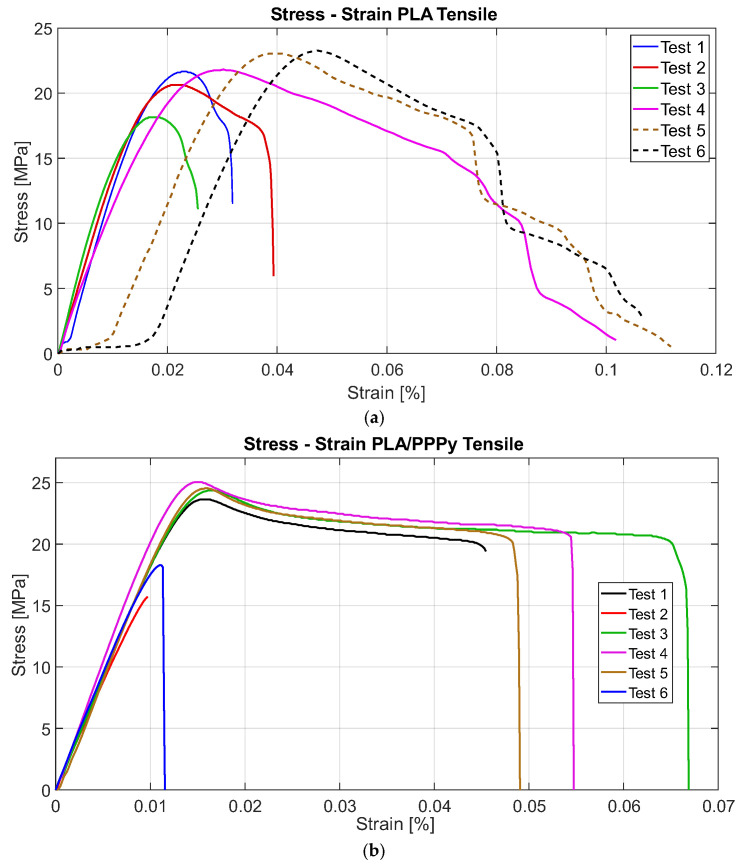
Tensile tests, (**a**) PLA tests, and (**b**) PLA/PPPy coating tests.

The first block of pure PLA tests exhibits similar behavior in only half of the tests, as the PPPy coating shows a significant correlation between each test. Using the collected load and displacement data, it is possible to reconstruct the stress–strain diagram, which is sufficient to divide the load by the area of the transversal section and the displacement between the initial length. Approximately 300 data points were analyzed for each test, and their distribution reflects the test behavior. Figure 9 describes all test behavior for PLA and PLA/PPPy coating tensile tests.

**Figure 10:**
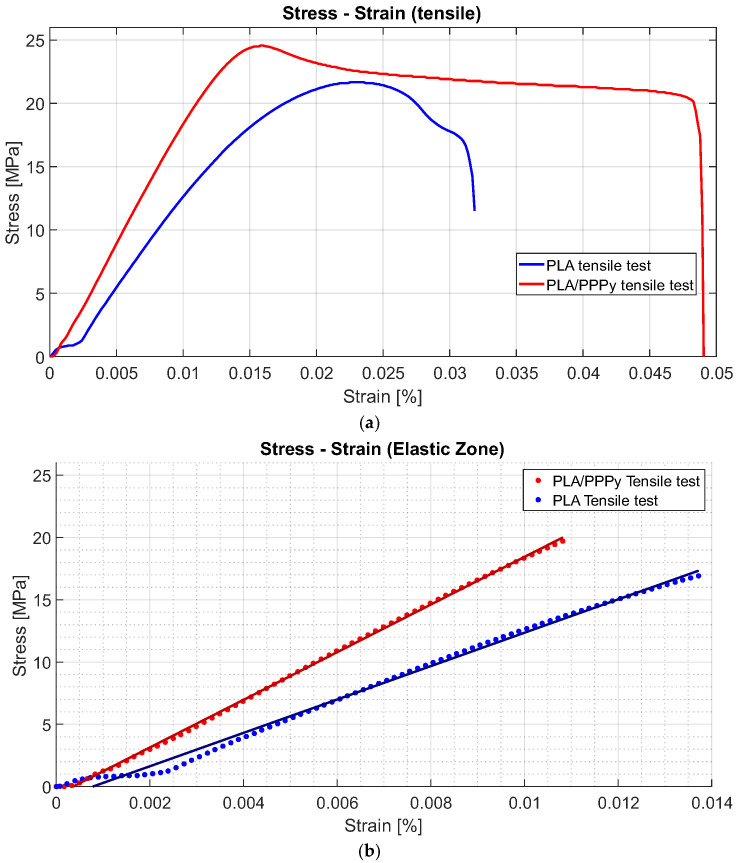
Comparative tensile tests for PLA and PLA/PPPy samples, (**a**) Complete test and (**b**) Elastic zone behavior.

From the stress–strain diagrams shown above, a similarity or repeatability in the precision of the tests can be appreciated. Some properties, such as the elastic limit, the ultimate stress, and the proportionality limit, can be extracted from the tests. The analysis of the elastic section of the test (the linear part of the diagram), taking the value of the proportionality limit as a significant point, allows a trend line to be linearized between all the points of that analysis segment (linear regression). With this, it is possible to estimate the value of the slope, which is also defined as the elastic modulus of the material. The difference between the elastic moduli of the PLA and PLA/PPPy samples results in the value of the approximation of the elastic modulus of PPPy only in the elastic zone of the test. Figure 10 describes an example of the difference between the diagram Stress–Strain for PLA and PLA/PPPy coating samples and their linear zone to estimate their elastic moduli. Additionally, Table 2 presents the results and averages for several mechanical properties obtained from each tensile test.

**Figure 11:**
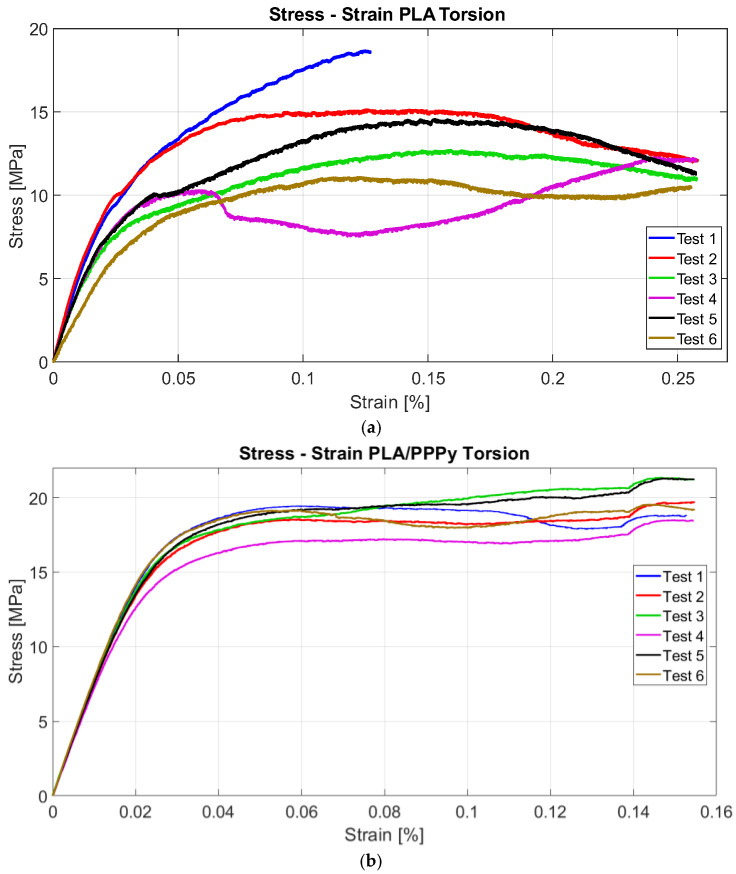
Torsion tests, (**a**) PLA tests, and (**b**) PLA/PPPy coating tests.

Similarly, all results obtained by the ASTM E143 method follow the same characteristic to estimate the shear modulus. The first group of PLA tests exhibited similar behavior across all tests. Five samples had a similar response to the test, and the machine recorded at least 20,000 data points for each test, including angle and torque values. It was necessary to estimate shear stress and Strain with mathematical expressions 1 and 2. Similar to the tensile test, the value of behavior on the torsion test increases when a layer of PPPy coat is applied to a PLA sample; Figure 11 illustrates this behavior for each test.

**Figure 12:**
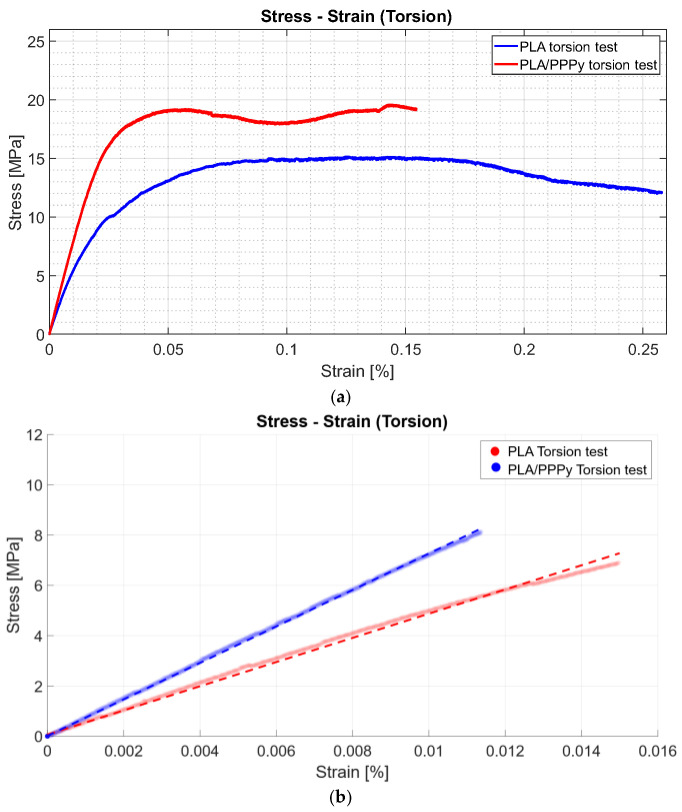
Torsion tests, (**a**) PLA and PLA/PPPy difference, and (**b**) Shear modulus.

The stress–strain diagrams shown previously exhibit a significant discrepancy between PLA tests and PPPy coating; in other words, this behavior is variable between them, rather than representing repeatability, which describes considerable control between tests. Just like tensile tests, some properties can be obtained from the diagram projection. The shear limit can be taken as a point for the elastic zone and used to estimate the value of the shear modulus as a linear function. The layer of PPPy on the PLA sample increases the value for mechanical behavior on the torsion test, and the linear function describes this increment in shear modulus. Figure 12 provides a comparative example, and Table 3 integrates the shear stress and shear modulus for each test, as well as the average and standard deviation for PPPy shear stress.

**Figure 13:**
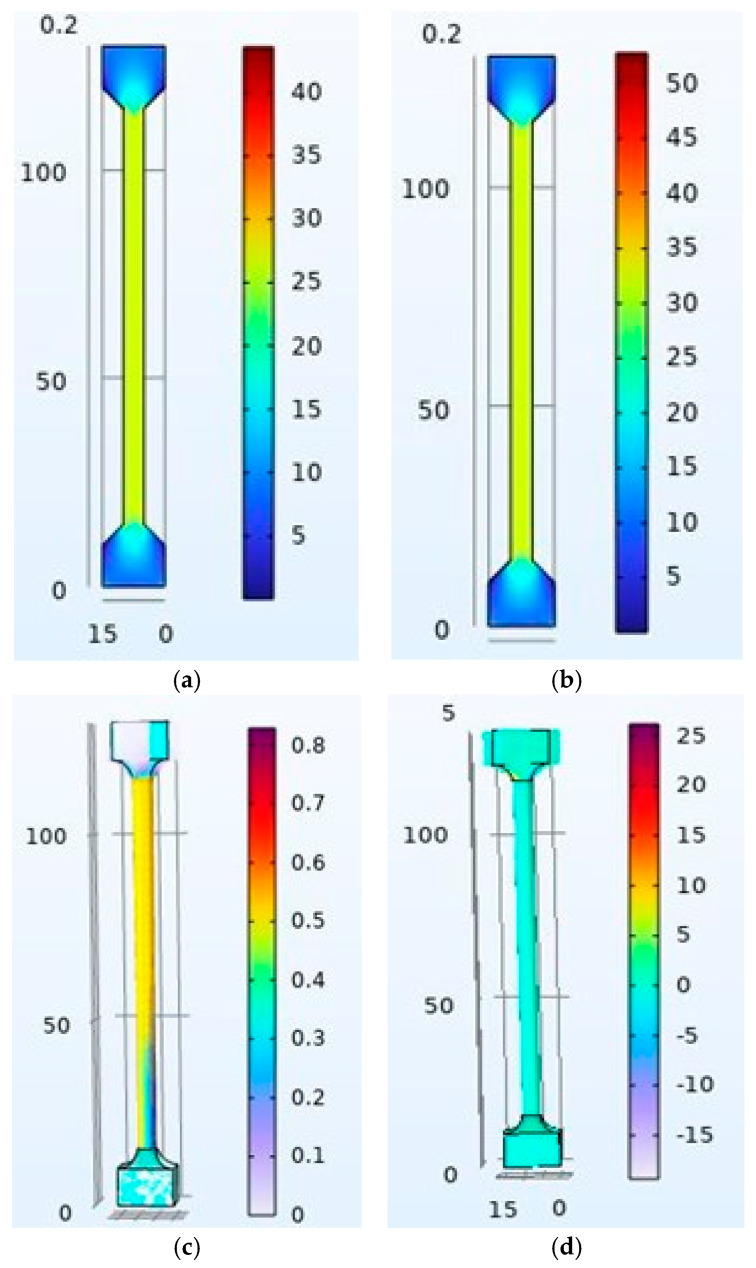
Stress and shear numerical results: (**a**) Tensile test for PLA, (**b**) Tensile test for PLA/PPPy, (**c**) Torsion test for PLA, and (**d**) Torsion test for PLA/PPPy, where all magnitudes are expressed on MPa.

**Figure 14:**
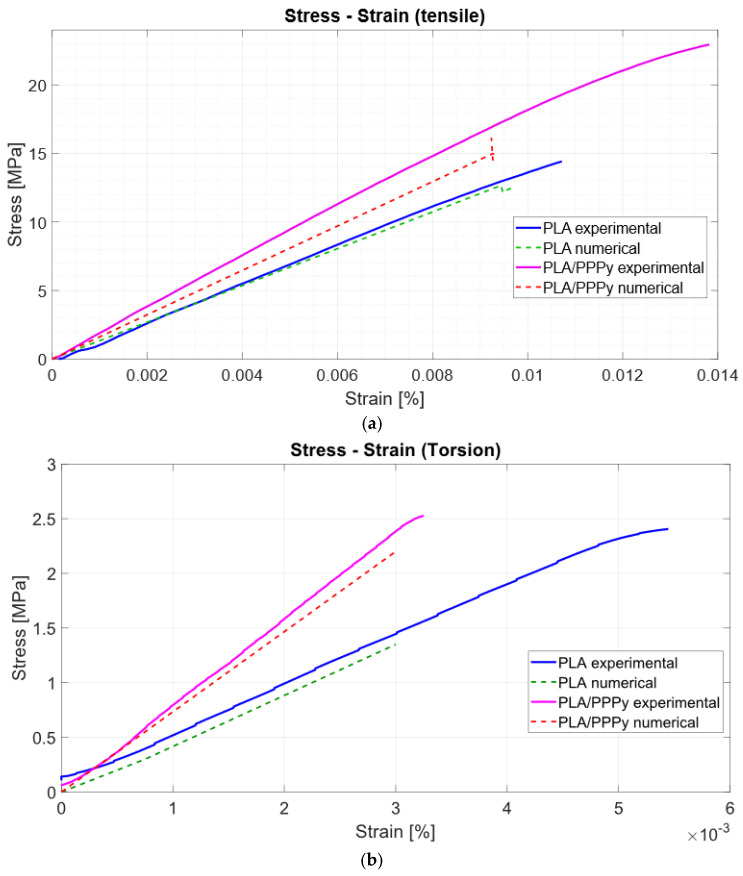
PLA Stress–Strain experimental and numerical diagram: (**a**) Tensile and (**b**) torsional.

For numerical tensile and torsion tests, we developed PLA and PPPy coatings using the computational tool COMSOL Multiphysics, according to PLA parameters such as density (1240 kg/m^3^), elastic modulus obtained by experimental tests as an average for all tests (1477 MPa) and PPPy coating (1715 MPa for elastic modulus and 1263 kg/m^3^ for density). Some studies have reported values for Poisson coefficient (PR) using the synthesis method. An electrochemical example is the research carried out by Amy [24], where the value for PR was 0.25, and in the research carried out by Della Santa [25], even though synthesis was not defined, it was reported that dopant for the samples increased the value for PR to 0.4629. Since plasma oxidation has not been reported for PR, the intermediate value was taken from simulation development (0.35). It was necessary to scale tensile and torsion models to include PPPy thickness. This contribution addresses the primary issue of thickness and support geometry. If considered as a material phase, all results describe the behavior of the material for the central mass. The relationship between the stress–strain diagram and the difference from experimental data for each test is described in Figure 13 and Figure 14.

For the statistical analysis, it was necessary to calculate the average value for each group of tests, their standard deviation, and the variability coefficient, which is defined as the ratio between these two parameters. As mentioned before, four groups of 6 samples were developed; however, only the first group did not consider all tests satisfactory. This group consisted only of tensile samples for PLA. Repeatability behavior was observed in only 4 out of all tests. For the following groups, this effect does not repeat in subsequent tests. The results of the statistical analysis are described in Table 4.

## 4. Discussion

There are many ways to estimate and validate the mechanical properties of materials. However, difficulties arise in some cases, particularly in thin films of polymers, where the thickness of the material or the total production required to develop a correct sample makes it challenging to select the appropriate experimental method to characterize the material’s elemental mechanical properties. For example, the main shortcomings and complications of this research stem from the lack of tensile testing on pure polypyrrole specimens and the absence of a support element, such as PLA. This is because, in the production of the polymer, it does not meet the necessary length requirements to perform the test based on the test methods. Recently, researchers have designed proposals to evaluate and identify the mechanical properties of materials, even when focusing on determining other properties, such as electrical. To explain and discuss the complexity of our study about similar works, there are contrasts whose results validate their similarity concerning the following: firstly, with support PLA elements, the elastic modulus of the PLA samples agrees with those reported by Martínez’s research [26], where those values range from 0.35 to 3.5 GPa. The difference in our elastic modulus results primarily from the use and design of the test sample. Particularly, the length and thickness, due to printing configurations, can leave empty spaces in the sample’s fill, increasing the likelihood that the adhesion of the filament layers upon extrusion could weaken the sample and lower the elastic modulus value.

Regarding the characterization of mechanical properties method, nanoindentation is the most used process to evaluate properties; however, it may be more expensive than the studies proposed in this research. Additionally, mechanical properties are evaluated using a practical power function, as demonstrated by Vladimir’s research [27]. In this case, thin films of organosilicons were evaluated with current variations. As a result, they report values of elastic modulus for the effective power response. In contrast, in this research, a constant current of 30 W was induced on the PLA sample. The elastic modulus values showed a similar behavior across all tests. The presence of polymer, specifically poly pyrrole, in tissue engineering demonstrates its adaptive and advantageous properties for implementing in organs and optimizing connections, as well as improving neuronal functions. For example, there exists an evaluation of Julio’s research [28], which proposes the development of tubular scaffolds for PLGA (polylactic-co-glycolic acid) and then doping with plasma PPy for use in urethral tissue engineering. In this research, the mechanical properties of scaffolds were evaluated using a tensile test after doping with PPy. Define dimensions for tensile samples, but do not specify whether they were according to standards or norms.

Additionally, they report values of elastic modulus before the doping process but do not specify the number of samples or tests. The importance of evaluating length for transversal sections has a direct relation to the rate value, and consequently, the test velocity condition. Differences between the last-mentioned are addressed in this article to offer reliable results and alternatives for characterizing the mechanical properties of polymers that production may find challenging to develop tests for.

## 5. Conclusions

Alternatives to polymeric materials, such as poly pyrrole, whose physical, chemical, and biological characteristics do not compromise or impair its interaction with tissues, represent a novel solution and field of research. Poly pyrrole was first used in neurological regeneration and has shown promising advances in recovering motor and sensory functions. The advantages of accessibility, synthesis for plasmatic oxidation, and compatibility make it an excellent option for implanting and use in tissues whose function is compromised by injury. It has shown that all variations in the elastic modulus of poly pyrrole are directly influenced by the synthesis methodology by which it is produced; in the chemical process, the elastic modulus is in the range for 2 or 3 GPa when it is used as a composite on mixed with another material; in the case of the electrochemical process, it is near the 20 or 50 kPa range; with this alternative for estimating mechanical properties for poly pyrrole produced by plasma oxidation, the result has a value near to 360 MPa only for the polymer. The custom design of tensile samples for use in the characteristic tests considered the area of the grips and their attachment, ensuring a proper fit and alignment without compromising the test area. Additionally, the post-processing for each sample could make a difference in test behavior. In PLA samples, all characters were guaranteed; however, when tensile or torsional tests were performed, the relationship between stress and Strain behavior varied depending on the test performed on the same sample. This effect can be attributed to the slight increase in temperature during the polymerization process, which likely led to fusion or significant adhesion in the PLA and PPPy samples, thereby enhancing their mechanical behavior in the test. As future work, it is considered important to evaluate the polymeric adhesion level on the surface. Not only can the polymer stabilize the stress and Strain, but it is also necessary to carry out more tests on PLA to ensure greater repeatability. Analysis of the elastic zone in the stress–strain diagrams of pure PLA and poly pyrrole-coated samples reveals a clear visual difference between the test slopes. The value of the tensile modulus estimated graphically using linear regression considers the adjusted behavior of the material’s elasticity by integrating the data series into a linear model whose slope accounts for all the points surrounding it. For the elastic section, it is possible to approximate the value of the elastic modulus. For PPPy, the value is 360.11 MPa. This result was evaluated using the computational tool COMSOL Multiphysics to validate each test. The similitude for each modulus is based on the origin of plastic deformation in the torsion sample caused by the constant current during polymerization; therefore, the value for the shear modulus is similar in both numerical and experimental tests.

## Figures and Tables

**Table 1:** Material properties for numerical test.

Sample	ρ [kg/m^3^]	E [GPa]	E.L. [MPa] *	*v*
PLA	1240	1.477	17	0.35
PLA/PPPy	1268	1.780	20	0.35

* E.L. Elastic limit and E Young Modulus.

**Table 2:** Experimental Tensile modulus on PLA and PLA/PPPy samples.

Sample	E.L. [MPa] *	L.S. [MPa] *	E [MPa] *
PLA	17	22	1349.7
	18	21	1392.7
	14	23	1689.3
	19	17.5	1342.6
Average	17	20.87	1443.57
SD			184.86
PLA/PPPy	20	24	1715.2
	15	16	1662.6
	23	24.5	1772.6
	24	25	2002.9
	23.5	24.5	1884.1
	17.5	18	1784.7
Average	20.5	22	1803.68
SD			122.74
PPPy	3.5	1.13	360.11

* E.L. Elastic limit, L.S. Last Stress, and E Young Modulus.

**Table 3:** Experimental Shear modulus on PLA and PLA/PPPy samples.

Sample	E.L. [MPa] *	L.S. [MPa] *	G [MPa] *
PLA	9	18.5	383.94
	9.5	15	396.02
	7	14.3	325.26
	7.5	13	356.36
	7.8	10	370.11
	6	11	258.68
Average	7.8	13.63	348.39
SD			50.30
PLA/PPPy	15	19	718.94
	16	18.5	708.33
	17.5	18	707.07
	12.5	16	739.39
	14	16.5	725.53
	16	16	655.22
Average	15.16	17.33	713.58
SD			50.26
PPPy	7.36	3.7	365.19

* E.L. Elastic limit, L.S. Last Stress, and G Shear Modulus.

**Table 4:** Variability coefficient value for tensile and torsion tests.

Sample	Average E [MPa]	Standard Deviation	Variability Coefficient *
PLA *	1477	184.86	13.27
PLA/PPPy *	1803.61	122.74	6.9
PLA	348.39	50.30	13.84
PLA/PPPy	688.7	50.26	7.1

* It refers to statistical values for the tensile test, and the other for the torsion test.

## Data Availability

The raw data supporting the conclusions of this article will be made available by the authors on request.
